# Supplementary material for: Calcium and phosphorylation coordination is a novel mechanism that stabilises protein-complexes during HIV assembly
Source: J Biomed Sci. 2026 Jul 2;33:70. doi: 10.1186/s12929-026-01274-7 (PMC13326380; doi:10.1186/s12929-026-01274-7)
Supplement: Supplementary file 1 — Supplementary material1. [file 12929_2026_1274_MOESM1_ESM.docx]

**
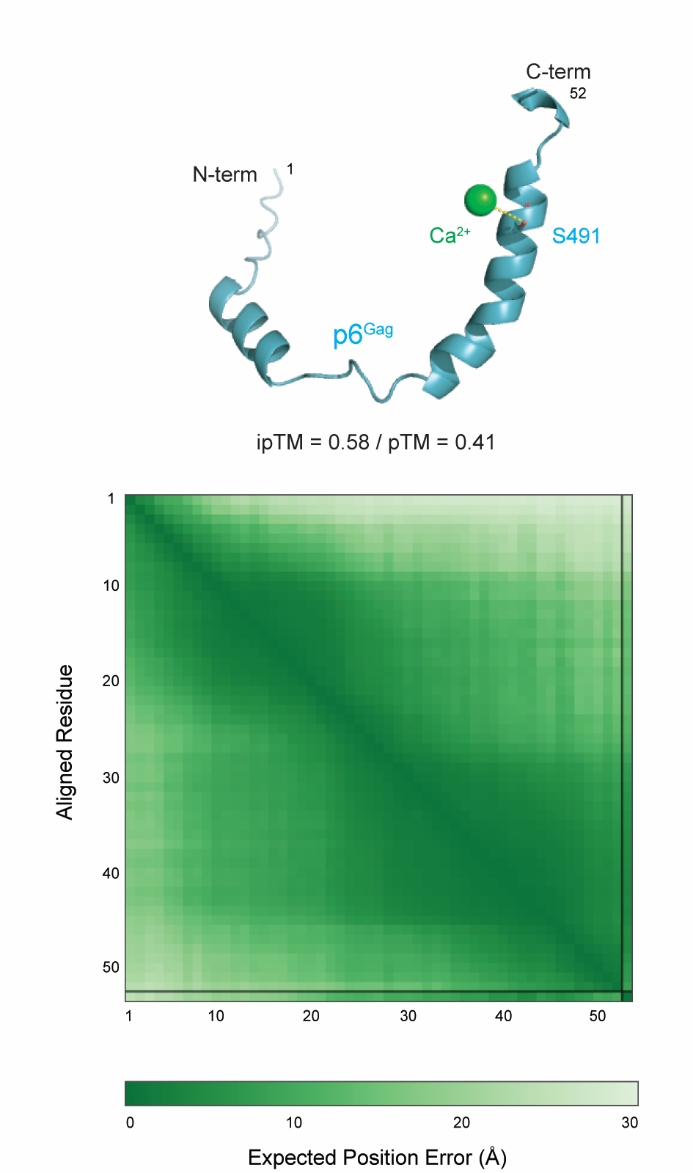
**

**Supplementary Figure 1: AlphaFold 3 predicted p6^Gag^-Ca^2+^ complex suggests Ca^2+^ likely binds p6^Gag^ near S491 residue.**

AlphaFold server reports that “PAE and/or pLDDT may be more indicative of prediction accuracy [whilst] pTM is less useful for small structures and short chains. This is because the TM score is very strict for smaller molecules”, hence average pLDDT > 70 is presented in Figure 1. Expected position error matrix also highlights high predicted position accuracy, particularly in both predicted alpha-helical domains, whilst ipTM = 0.58 / pTM = 0.41

**
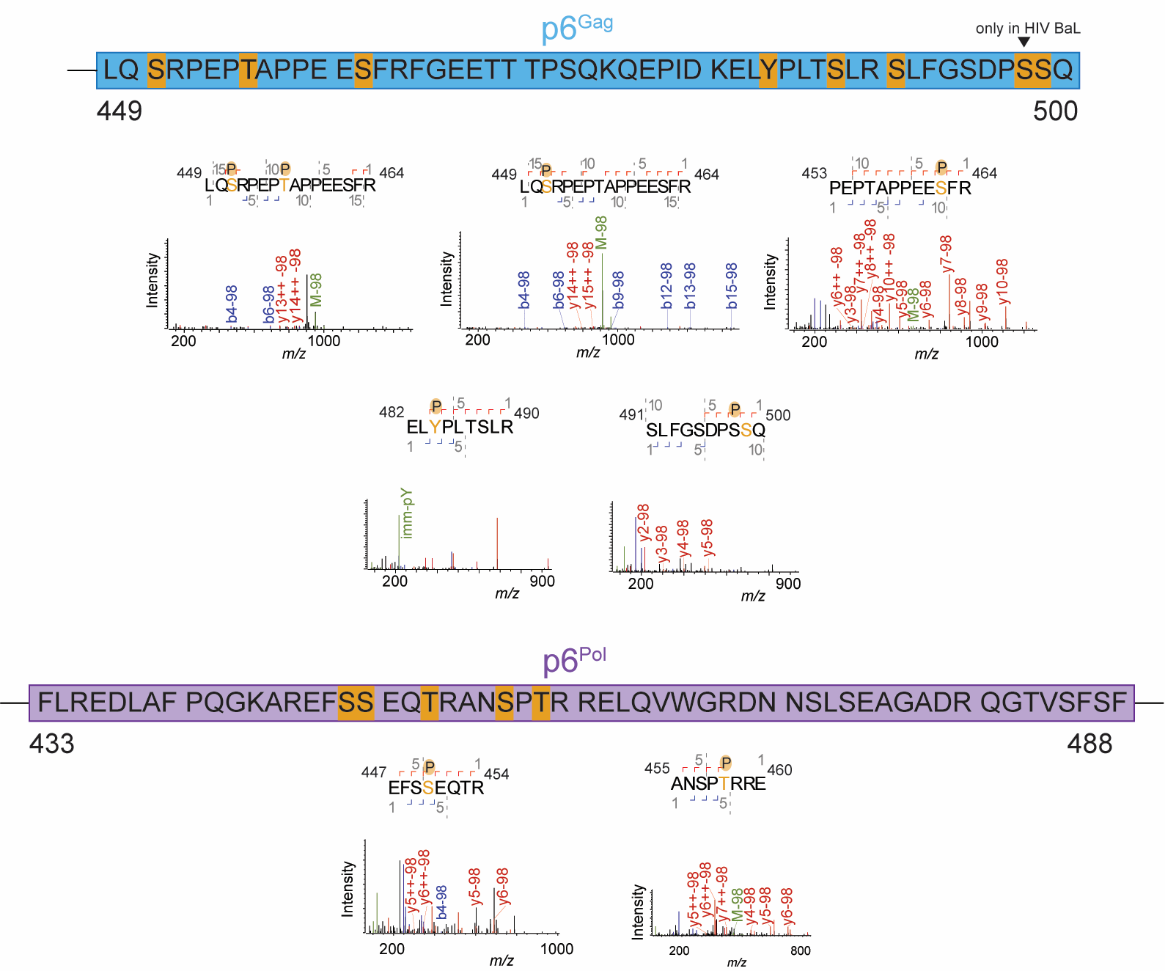
Supplementary Figure 2: Example of MS spectra displaying evidence of discovered/confirmed sites phosphorylation in p6^Gag^ and p6^Pol^.**

Large quantities of HIV BaL and NL 4.3 were lysed, and proteins were processed for phospho-proteomic analysis. Phospho-peptides enrichment was realised by hydrophilic interaction chromatography followed by metal affinity chromatography. Proteins subsequently underwent tryptic digestion for MS phospho-proteomic analysis. Phosphorylated sites in p6^Gag^ and p6^Pol^ were plotted with their occurrence frequency amongst Pr55^Gag^ and Pr160^GagPo^l phosphorylation events.

**
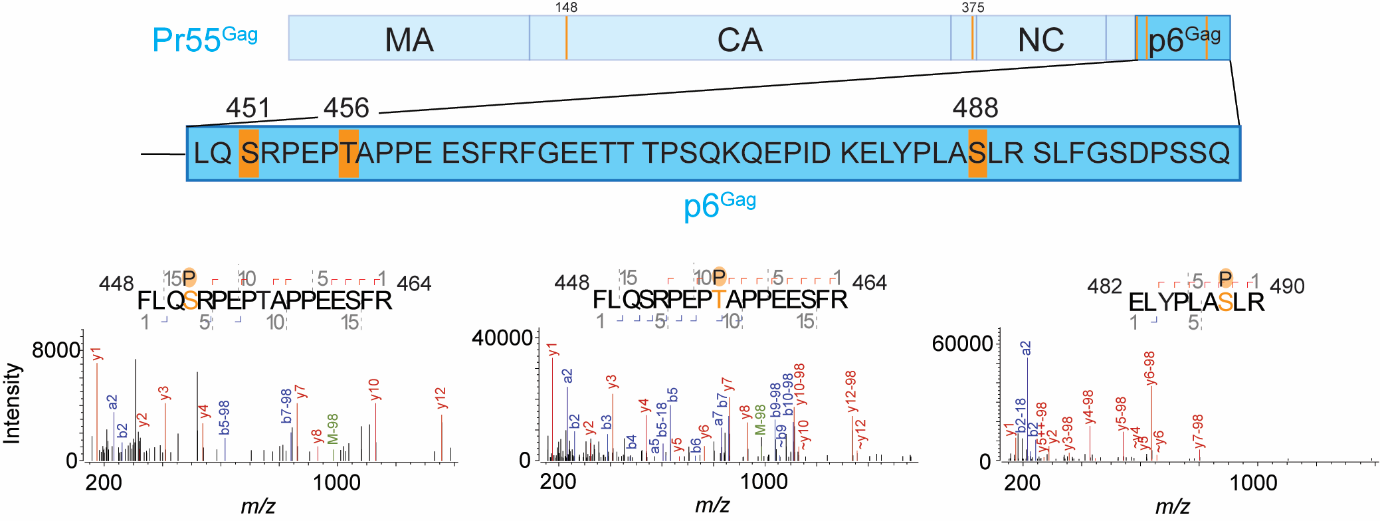
Supplementary Figure 3: Recombinant Pr55^Gag^ phosphorylation sites and levels upon *in vitro* phosphorylation using active ERK-2.**

Recombinant active GST-tagged ERK-2 was used for *in vitro* phosphorylation of Pr55^Gag^ (1 ng enzyme per μg of substrate) in a 1-hour reaction at 30ºC. Pr55^Gag^ was separated from ERK-2 by capture chromatography, and in-gel digested for MS phospho-proteomic analysis.

**
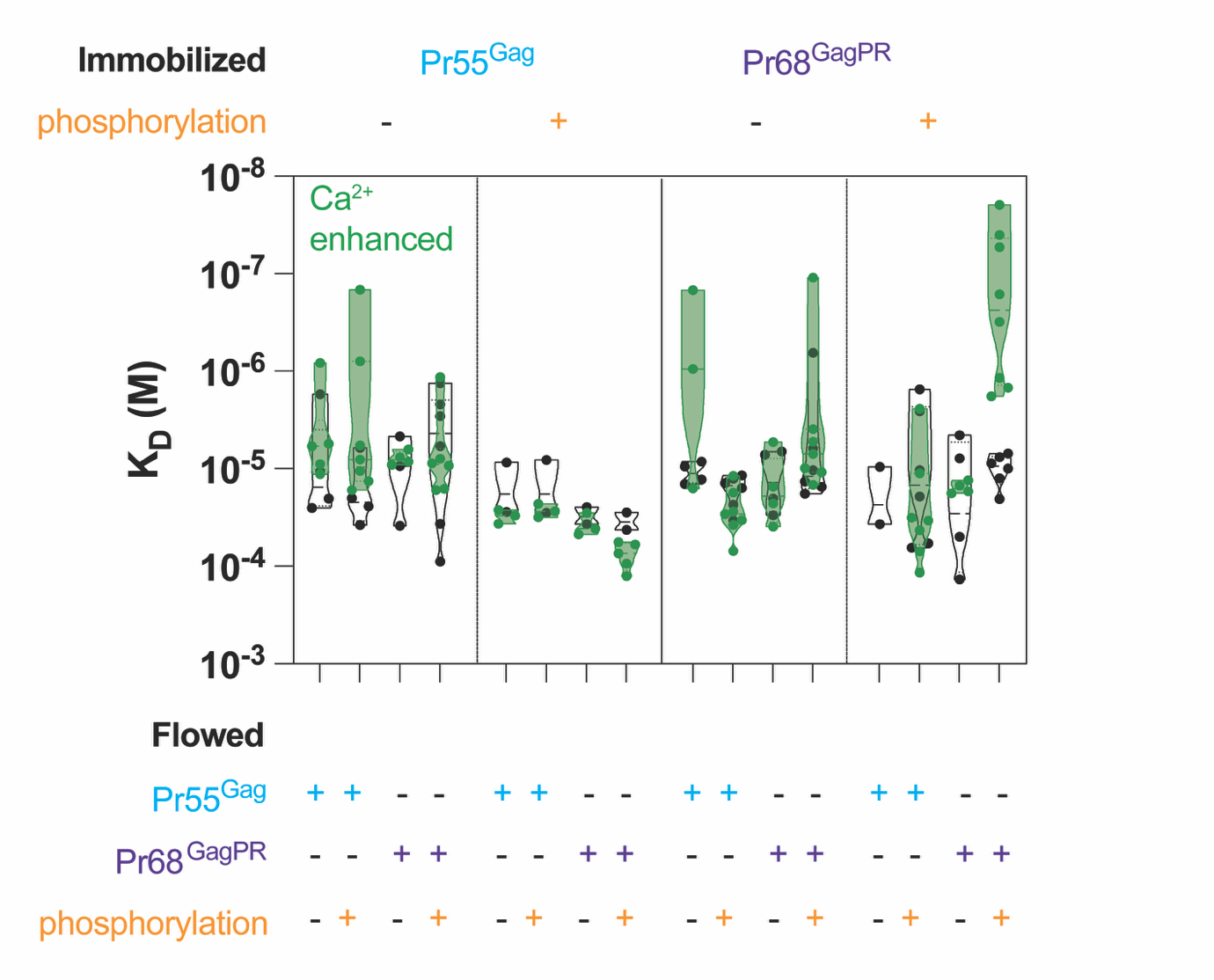
Supplementary Figure 4: SPR measurements of protein-protein interactions K_D_ with/without Ca^2+^ mediation.**

Pr55^Gag^, Pr68^GagPR^ and their phosphorylated versions were immobilized. An excess of Ca-acetate was flowed against immobilized proteins to prime subsequent protein-protein interactions with flowed Pr55^Gag^, Pr68^GagPR^ and their phosphorylated versions (green). Interactions KD values were compared between direct protein-protein interactions (black) and Ca^2+^-mediated protein-protein interactions (green).

**
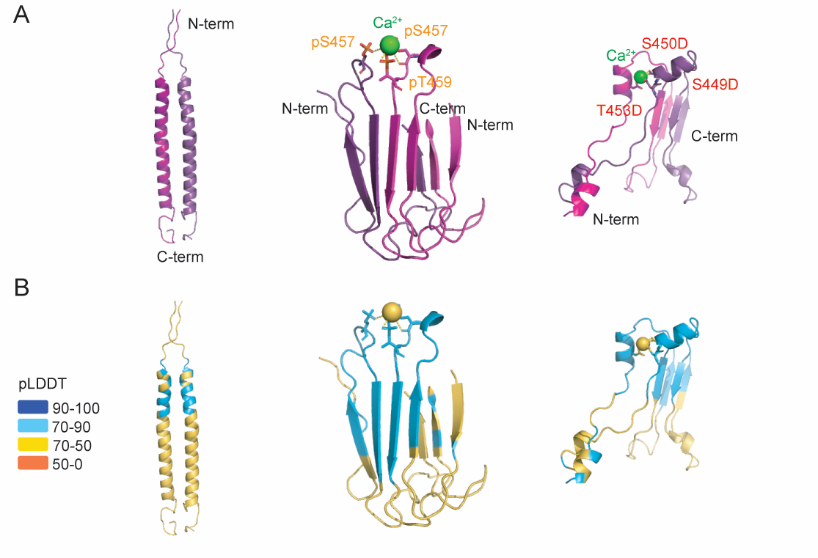
**

**Supplementary Figure 5: Ca^2+^-phosphate bridges in p6^Pol^ dimer predictions using AlphaFold 3.** (A) Predicted p6^Pol^ dimer structure compared to Ca^2+^ mediated phosphorylated or phospho-mimicking p6^Pol^ dimers. Five sites (S449, S450, T453, S457, T459) are phosphorylated / mutated into D in both p6^Pol^ peptides. (B) Local confidence level of AlphaFold 3 predictions have pLDDT value of > 70 for residues in regions covering S449-T459 in all three simulated dimers composed of 55 residues-long monomers.

**
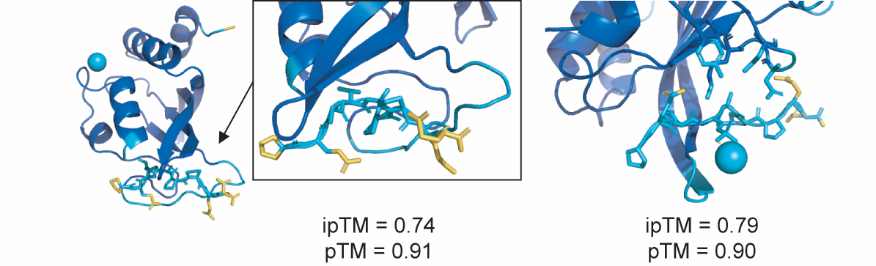
**

**Supplementary Figure 6: Influence of T456 phosphorylation on Tsg101 UEV domain complex with p6^Gag^ fragment containing PTAP predicted structure in presence of Ca^2+^ using AlphaFold 3.**

Influence of pT456 in PEPTAPPEE p6^Gag^ peptide on predicted complex structure with Tsg101 UEV in presence of Ca^2+^. Proteins structures and interactions predictions are trustworthy (ipTM ≥ 0.74 and pTM ≥ 0.90 in both predictions). Locally, pLDDT ≥ 70 near investigated residues in both predictions.


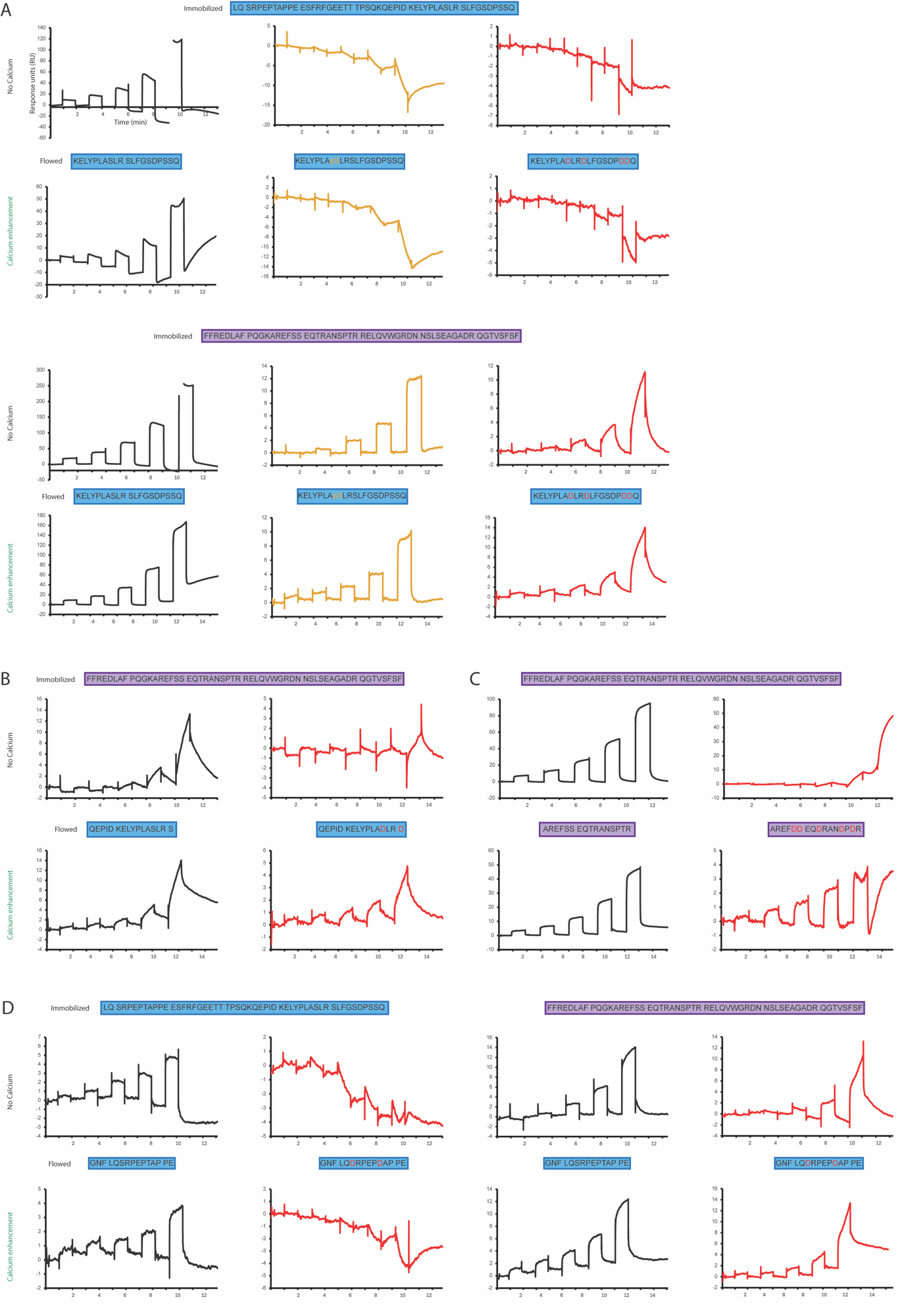


**Supplementary Figure 7: SPR chromatograms of peptides interactions with immobilized SP2-p6^Gag^ and p6^Pol^**

Full length biotinylated SP2-p6^Gag^ / p6^Pol^ were immobilized onto streptavidin chips. Peptides fragments derived from SP2-p6^Gag^ / p6^Pol^ and corresponding phosphorylated versions or phospho-mimics were flowed at concentrations ranging from 12.5 to 200 uM in single cycle mode. Response in RUs, time in minutes.

A) Interaction chromatograms (with or without Ca^2+^) of peptide WT (black), phosphorylated pS488 (orange), and phospho-mimic (red) interaction with immobilized SP2-p6^Gag^ / p6^Pol^. Flowed and immobilized sequences are presented, phosphorylation sites (alternatively phospho-mimics) are highlighted in colour, SPR data are presented in Figure 3B-C.

B) Interaction chromatograms (with or without Ca^2+^) of WT peptide (black) compared to phospho-mimic (red) with p6^Pol^. SPR data presented are from Figure 3D.

C) SPR interaction chromatograms of p6^Pol^ derived peptide (WT in black, phospho-mimic in red) with immobilized p6^Pol^. SPR data presented are from Figure 4C.

D) SPR interaction chromatograms of T456 containing peptides (WT in black, phospho-mimic in red) with immobilized SP2-p6^Gag^ / p6^Pol^. SPR data presented are from Figure 5C.
